# Social prioritisation in scene viewing and the effects of a spatial memory load

**DOI:** 10.3758/s13414-023-02769-3

**Published:** 2023-08-10

**Authors:** A. P. Martinez-Cedillo, Kevin Dent, Tom Foulsham

**Affiliations:** 1 Department of Psychology, University of York, York, YO10 5DD UK; 2 Department of Psychology, University of Essex, Wivenhoe Park, Colchester, Essex, CO4 3SQ UK

**Keywords:** Attentional capture, Eye movements and visual attention, Memory, Visual working and short-term memory

## Abstract

When free-viewing scenes, participants tend to preferentially fixate social elements (e.g., people). In the present study, we tested whether this bias would be disrupted by increasing the demands of a secondary dual-task: holding a set of (one or six) spatial locations in memory, presented either simultaneously or sequentially. Following a retention interval, participants judged whether a test location was present in the to-be-remembered stimuli. During the retention interval participants free-viewed scenes containing a social element (a person) and a non-social element (an object) that served as regions of interest. In order to assess the impact of physical salience, the non-social element was presented in both an unaltered baseline version, and in a version where its salience was artificially increased. The results showed that the preference to look at social elements decreased when the demands of the spatial memory task were increased from one to six locations, regardless of presentation mode (simultaneous or sequential). The high-load condition also resulted in more central fixations and reduced exploration of the scene. The results indicate that the social prioritisation effect, and scene viewing more generally, can be affected by a concurrent memory load.

## Introduction

The world in which human behaviour takes place is complex and cluttered, with many possible stimuli to analyse and act upon. According to current conceptualisations of attention, stimuli compete for access to neural and computational resources, which in turn govern access to behavioural responses (e.g., Desimone & Duncan, 1995). The question of how and why particular stimuli may be prioritised during this competition is an old one (e.g., see Moray, 1959), which has often focussed on the relative importance of physical stimulus properties, in comparison to the meaning that the stimuli hold for the observer.

Recently this debate has played out in the context of the question of which stimuli “capture attention” when we view scenes. According to one tradition, physical properties such as “saliency from feature contrast” are particularly important (see Theeuwes, 2010, for a review). For instance, high physical salience distractors tend to be targets for early saccades, even when the goal is to explicitly ignore them (Anderson & Donk, 2017; Donk & Van Zoest, 2008; Foulsham & Underwood, 2008; Underwood & Foulsham, 2006). Other research has emphasised the importance of the meaning of objects for predicting saccades. In particular, even when objects are of similar physical salience, participants tend to preferentially direct fixations to items of social significance (e.g., people; see Birmingham et al., 2008a, b, 2009; End & Gamer, 2017, 2019).

Recent research has begun to investigate the behavioural characteristics of this prioritisation of social objects. We recently investigated whether the social prioritisation effect was reduced when verbal working memory resources were taxed (Martinez-Cedillo, Dent & Foulsham, 2022). Increasing verbal working memory load is a technique known to disrupt goal-based control of attention in the face of a salient distractor (e.g., Boot et al., 2005; Burnham, 2010; Lavie & De Fockert, 2005). Our results showed that the social prioritisation effect was undiminished under the conditions of a high memory load, demonstrating the robust and perhaps obligatory nature of this effect.

Bianchi et al. (2020) also recently investigated the effects of a cognitive load on looking behaviour in real and depicted environments in which a confederate was present. Their results showed that looking towards the confederate was disrupted when participants were given a cognitive load. However, in this study the cognitive load task took the form of an n-back task in which participants were continuously presented with distracting information. Given the close links between auditory and visual spatial attention (e.g., see Spence & Driver, 1996), the disruption involved could well be occurring not because of a domain-general cognitive load, but because of specific interference with visual spatial attention. Furthermore, in the study of Bianchi et al. (2020), a cognitive load condition is compared against a condition involving no load, meaning that a high cognitive load is confounded with other more general dual-task scheduling processes. In contrast, the study by Martinez-Cedillo et al. (2022) compares low and high cognitive load conditions, meaning that only the amount of load differs between conditions, and other more general dual-task processes are held constant. In addition, in the study of Martinez-Cedillo et al., the scene-viewing task was restricted to the retention interval of the memory task, meaning that monitoring for memory stimuli would not disrupt scene viewing. Following on from our previous research, the question naturally arises just how impervious to interference the social prioritisation effect is. In this context, it is important to realise that not all secondary tasks are equivalent.

According to load theory (e.g., Lavie et al., 2003, 2004), cognitive load may be contrasted with perceptual load. Cognitive load refers to a load upon the central mechanisms responsible for maintaining task priorities or task sets (see Moore et al., 2017), and may be impacted by a verbal working memory load. In contrast, perceptual load refers to the load placed on the resources required for perceptual analysis and identification of stimuli. While perceptual load is often manipulated by increasing the number or complexity of the stimuli in a display (see Lavie & Cox, 1997; Lavie & Tsal, 1994), Lavie and colleagues have suggested that it may also be increased by a secondary task in which the to-be-remembered stimuli come from the same domain as the primary stimuli (i.e., visuo-spatial memoranda in the case of viewing visual scenes). In particular, Konstantinou et al. (2012), Konstantinou and Lavie, 2013, Konstantinou et al. 2014 have shown that a visuo-spatial memory load can act to reduce perceptual processing in a way similar to directly increasing the number of stimuli. Typically, increased perceptual load results in reduced capture by irrelevant stimuli, but also more generally reduced perceptual processing of presented stimuli.

In the context of manipulations of perceptual load using display complexity or display size, it has been shown that certain stimuli like faces (e.g., Lavie et al., 2003) or objects of high familiarity (e.g., musical instruments for expert musicians; Ro et al., 2009) can continue to exert interference when irrelevant, even in the face of high perceptual load. Thus, it is certainly the case that the processing of irrelevant stimuli may continue to proceed even in the face of a high perceptual load. However, measuring the perceptual load of primary task visual stimuli is not straightforward (for discussion, see Roper et al., 2013; Tsal & Benoni, 2010). By some measures, complex and cluttered scenes may be considered to already present a high perceptual load. In order to side-step the issue of measuring the perceptual load imposed by scenes, in the present study we manipulated perceptual load by virtue of a secondary visuo-spatial memory load. Will the social prioritisation effect in scene viewing show invulnerability to an increased perceptual load imposed by a competing visuo-spatial memory task?

Other authors have recently argued that looking at people in scenes is automatic and reflexive (e.g. Rösler et al., 2017), since it occurs without an explicit goal to inspect people, it occurs even for the very first saccades, and it occurs in response to briefly presented images. Aside from the control of eye movements, earlier work also demonstrates that participants are able to detect the presence of animal and human bodies, and faces in complex images both with very brief displays (Kirchner, & Thorpe, 2006; Thorpe et al., 1996) and with attention diverted (Li et al., 2002). Such rapid identification may occur on the basis of the “feedforward” processing courtesy of specialised structures tuned to detect real objects (e.g., Van Rullen, 2009) and their constituent features (e.g., Evans & Treisman, 2005). If people in scenes are detected rapidly, then a key question is whether participants will fixate them regardless of the current task demands. This provides one test of automaticity, in the sense that if participants cannot modulate their prioritization of social regions then it is automatic. Of course, other aspects of processing may be important for automaticity (such as how rapidly they occur), and tasks need not be thought of as dichotomously fully automatic or completely under voluntary control. Our investigation contributes to this by testing whether orienting to people in scenes will continue undiminished, even in the face of competing demands from a second demanding task presented in the same sensory domain.

The present study examined the effect of increasing visuospatial memory load on image viewing. Is the social prioritisation effect sufficiently obligatory and automatic as to persist in the face of competition from this type of load? It is possible to think of the competition imposed by the working memory task as competition for shared perceptual processing resources. According to this view, increasing visuo-spatial working memory load would result in increased perceptual load, and would withdraw perceptual processing resources from the scene viewing task (see Konstantinou et al., 2012, 2014, for behavioural and neuroscientific evidence). To the extent that the explicitly relevant memory task outcompetes the scene-viewing task for any perceptual resources required to drive social prioritisation, this prioritisation should be diminished. In contrast, if either the social prioritisation effect does not depend on perceptual resources that overlap with the memory task, or the priority for processing social items is set so high as to outcompete the memory task, we may anticipate no effect.

An alternative way of conceptualising the competition that may occur between the two tasks is in terms of more direct competition to the distinct locations occupied by the stimuli in the two tasks. Retaining spatial information in memory may impact shifts of spatial attention directly, as eye movements to remembered locations may be recruited in the service of memory rehearsal (e.g., see Baddeley, 1986). Certainly, it has been known for some time that eye movements to locations that are incongruent with to-be-remembered locations result in interference with memory (e.g., Hale et al., 1996; Lawrence et al., 2004; Postle, 2006; Pearson & Sahraie, 2003). In addition, participants often make spontaneous eye movements to the locations of to-be-remembered stimuli, even for stimuli where the location is not explicitly relevant (e.g., Richardson & Spivey, 2000). However, it is not clear that these location-congruent eye movements are functional in the sense of improving memory. It is certainly the case that participants can maintain a high level of spatial memory even when fixation is held constant during a retention interval. For example, Smyth and Scholey (1994) showed that memory for a sequence of locations in the Corsi Blocks test was no different when participants were free to move their eyes in the retention interval, compared to when fixation was held constant (see also Godjin & Theeuwes, 2012, for a similar result). However, it is important to note that under these conditions shifts in spatial attention and eye-movement planning remain possible. The deleterious effect of irrelevant incongruent eye movements is then explained not by the eye movements per se, but by the shifts of spatial attention that typically accompany them. Thus, one possibility is that in the face of a high spatial working memory load participants will make fewer eye movements, and correspondingly fewer shifts of covert spatial attention to the scene during the retention interval in order to protect memory. Our question is then if the eye movements that underly the social-prioritisation effect persist even though they may impose a cost to memory.

Research shows that if eye movements to memoranda during the Corsi Blocks task are not merely suppressed but rendered impossible by presenting the stimuli to participants with their head and eyes turned in opposite directions, such that the eyes cannot rotate any further to fixate the stimuli, there are costs to spatial memory performance (Ball et al., 2013; Pearson et al., 2014). However, here both actual eye movements, eye-movement planning, and exogenous deployments of attention (see Pearson et al., 2014) are all disrupted. These studies serve to underline the importance of possible eye movements and shifts of attention to relevant stimuli during the Corsi Blocks task.

Regardless of how one conceptualises the competition between the two tasks either mediated by perceptual load or more directly in terms of competition between the stimulus locations involved, the question remains whether the social prioritisation effect is sufficiently obligatory and automatic to remain undiminished in the face of such competition. The current study sought to answer this question.

A previous study by Cronin et al. (2020) compared the effects of a visual and a verbal memory load on scene viewing in general but did not specifically examine social prioritisation or the fixation of particular regions. The results showed that both a visual and a verbal memory load altered viewing patterns in the scene-viewing task, such that participants increasingly fixated on the centre of the screen. However, like the study of Bianchi et al. (2020), a no-load control was compared against a high-load condition, meaning it was impossible to separate the specific effect of increasing memory load from the more general effect of carrying out a second task at all. The current work went beyond that of Cronin by specifically comparing a small and large visuospatial memory load and specifically investigating the social prioritisation effect.

One important consideration regarding spatial working memory is the mode of presentation. Most early studies presented locations sequentially and asked participants to recall the items in the correct order so as to aid comparisons with verbal working memory, which is more naturally sequential. A number of studies have compared memory for simultaneously presented matrix patterns (“pattern span”) with variations of the Corsi Blocks task in which participants attempt to recall sequentially presented locations, in the correct serial order. Della Salla et al. (1999), for example, showed that these two tasks showed distinct patterns of interference. Other research points to distinct developmental trajectories for these two tasks (e.g., Logie & Pearson, 1997; Pickering et al., 2001). Critically, recent research using the abducted eye paradigm, in which saccades and exogenous shifts of spatial attention are rendered impossible, showed that sequential spatial memory tasks were interfered with but simultaneous spatial memory tasks were not. These data thus suggest that sequential tasks overlap to a greater extent with mechanisms for shifts of attention and eye movements. Recent research (e.g., Cortis Mack et al., 2015; Gmeindl et al., 2011) shows that even when serial order is not required participants tend to recall spatial locations in the order in which they were presented, at least for short lists of locations. Thus, whether or not participants are required to retain sequential spatial locations in order or not may make little difference to the underlying mechanisms involved at least for short lists of locations. On balance, previous research suggests that sequentially presented sets of spatial locations likely recruit shifts of spatial attention as a rehearsal mechanism to a greater extent than simultaneously presented sets of locations. Simultaneously presented sets of locations, in contrast, likely recruit configural representations that capture the overall form of the set of locations presented (e.g., see Gmeindl et al., 2011; Jiang et al., 2000).

Given the potentially greater role for spatial attention in maintaining sequentially presented spatial locations in memory, we compared simultaneously and sequentially presented spatial locations. If any disruption of the social prioritisation effect can be tied to competition for spatial attention and associated fixations, rather than more general perceptual resources, then disruption should be greatest in the sequential presentation condition.

## Method

### Participants

Sixty participants (ages 18–32 years, *M = 21.6 SD = 4.85* years; 42 females, 18 males) from the University of Essex took part in the experiment. Half of these participants took part in the sequential condition and half in the simultaneous condition, which were run at separate times. After discarding data from 13 participants who were not accurate in the calibration (mean error above 0.8°, a threshold set a priori) the sample size was 47. All participants reported normal or corrected-to-normal vision. Participants were paid £4 or received course credit for their participation.

### Apparatus and stimuli

The experiment was programmed in MATLAB (version 9.1.0, R2016b; the Mathworks, Natick, MA, USA), using the Psychophysics Toolbox. Eye position was recorded using the SMI RED500, which is a screen-based eye tracker that samples pupil position at 500 Hz. A 9-point calibration and validation were repeated several times to ensure that all recordings had a mean spatial error of better than 0.8° (mean across participants = 0.53°, SD = 0.27). Head movements were restricted using a chin rest. The experiment took place in a dimly illuminated, sound-attenuated room. Participants sat 60 cm away from the screen (a 22-in. CRT monitor) so that the stimuli subtended approximately 43° by 28° of visual angle at 1,680 × 1,050 pixels. The stimuli were a set of 64 high-resolution colour photographs, which were found from free access image databases, for instance Pixabay. Thirty-two of these were filler photographs that we did not analyse further but were used to divert attention from the experimental images. The fillers were taken from a similar set of images but did not contain the key objects of interest. The remaining 32 experimental images were selected to contain a pre-specified area of interest on opposite sides of the image: a social area (a person) and a non-social area (an object). We created two versions of each image using PicMonkey, an online image editing software, by manipulating the non-social object so that it was either high or low in visual saliency. These manipulations made the object stand out from its background to a greater or lesser degree, for example by changing the colour, and this was confirmed by using an implementation of the Itti and Koch saliency map model (Itti & Koch, 2000) via MATLAB (version 9.1.0, R2016b). This model selected the high-saliency object early (the first three simulated fixations) and the low-saliency object on later simulated fixations. Further details of these stimuli can be found in Martinez-Cedillo et al. (2022).

### Procedure

**Fig. 1:**
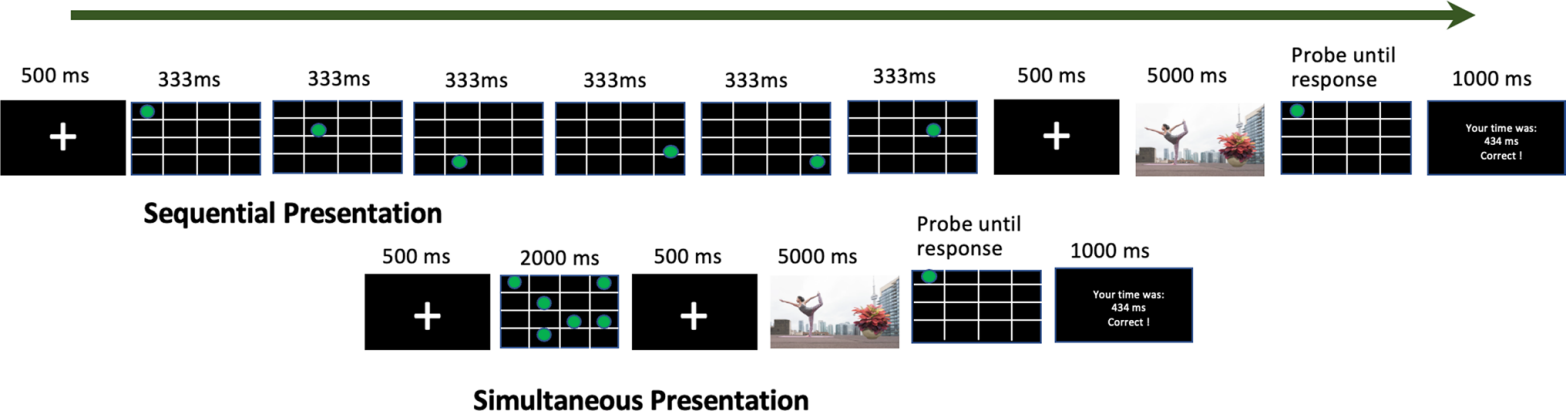
Experimental procedure showing the sequential (**above**) and simultaneous (**below**) memory task. Both examples show a trial from the high-load condition

Figure 1 summarises the experimental procedure. Participants performed a visuospatial working memory task where they were required to remember a set of dots. While doing this, they viewed each of the scenes.

The calibration and validation of the eye tracker were performed at the start of the experiment. The working memory task was adapted from McNab et al. (2008). Each trial started with a fixation dot displayed for 500 ms. After this, a 4 × 4 grid was displayed with either one (low load) or six (high load) dots presented in different locations on the grid. The dots were green on a black background.

In the sequential presentation condition, dots appeared one at a time (in high load). Each location was presented for 333 ms before disappearing and being replaced by the next location. In the low-load condition, the single location was presented for 2,000 ms. These timings were designed to ensure that the total presentation time was the same in both high- and low-load conditions (see McNab et al., 2008). In the simultaneous presentation condition, all the dots were shown on the screen at the same time for 2,000 ms. In all cases, the locations were chosen at random with no location repetition and followed by a fixation point presented for 500 ms.

Next, the scene was shown for 5,000 ms. Participants were instructed to look freely at the scene while performing the memory task. After each scene, a probe display was presented, and participants were required to indicate whether the probe location was one of those presented previously (or not) by making a keyboard response. After each response, a feedback screen was displayed, showing the reaction time and accuracy from that trial. Participants were encouraged to respond as quickly and accurately as possible.

The experiment consisted of two blocks with low (one dot) or high (six dots) memory load. Each block consisted of 32 trials (half fillers and half experimental images). Half of the participants started with the low-load block and half with the high-load block. Trials were equally divided between those with high and low saliency objects, and between those where the memory probe was present or absent. Experimental images were counterbalanced across participants such that each scene appeared in all load and saliency conditions, and scenes were also mirror reversed for half the participants to control for any biases to the left or right of the image. The experiment took approximately 25 min.

### Data analysis

Participants who scored below 50% on the memory task were excluded from the analysis. Trials with incorrect memory responses, and those in which the fixation at the onset of the scene was not on the centre, were also removed. Fixations were removed if their duration was below 100 ms. Following these criteria, we analysed data from 44 participants (ages 18–32 years, *M = 21.43, SD = 4.78* years; 34 females, ten males; 20 in sequential and 24 in simultaneous). For our main analyses, we compared participant means using a mixed ANOVA with the factors of load (high and low) and object saliency (high and low) and a between-subjects’ factor of presentation type (sequential and simultaneous). A sensitivity analysis indicated that our sample size allows us to detect medium effect sizes (Cohen’s *f* = 0.65) with a power of at least 95% when assuming a correlation of *r* = .50 between factor levels.

## Results

We examine the effects of encoding visuospatial information on the image-viewing task. We first report behavioural data from the memory task and general eye-movement behaviour. Then, we examine the effects of load on fixations to social and non-social elements.

### Behavioural data

Accuracy in the memory task was lower in the high-load condition *(M* = 78.56%, *SD* = 21.47) than in the low-load condition (*M* = 90.93%*, SD* = 11.09). A paired-sample t-test compared the accuracy of the memory probe under high and low loads*, t* (43) = -3.419,* p* < .001*,* confirming that the high-load task was more difficult.

### General eye-movement statistics

To investigate whether load interfered with eye movements, we examined the number and duration of fixations and the degree to which they showed a bias to the centre of the screen. This was compared across load and saliency conditions, but also across the two different types of presentation since we reasoned that controlling the sequence of the dots (sequential condition) might have an impact on eye movements during memory retention. Table 1 summarises these general eye-movement statistics.

**Table 1:** Measures of general viewing behaviour across conditions. Values show the mean across participants, with standard deviation in parentheses

	Load	High load		Low load	
	Saliency of non-social object	High	Low	High	Low
	Presentation type				
Number of fixations per trial	Sequential	18.49 (6.12)	16.75 (6.52)	19.06 (5.24)	18.54 (6.63)
	Simultaneous	17.15 (7.88)	16.67 (5.61)	17.03 (6.58)	17.64 (5.04)
Average fixation duration (ms)	Sequential	164.76 (64.00)	239.32 (117.42)	172.66 (43.40)	232.91 (100.59)
	Simultaneous	165.97 (67.36)	162.66 (62.70)	164.76 (64.00)	172.66 (43.40)
Average distance to the centre (degrees)	Sequential	11.19 (1.7)	11.31 (1.91)	11.29 (2.89)	11.97 (3.35)
	Simultaneous	10.55 (3.25)	10.12 (2.7)	11.46 (2.45)	11.89 (1.85)
Heatmap entropy (bits)	Sequential	18.91 (0.56)	18.82 (0.68)	19.24 (0.79)	19.43 (0.64)
	Simultaneous	18.68 (0.79)	18.72 (0.67)	19.45 (0.83)	19.54 (0.70)

Although there were some small differences between the number of fixations per trial and the average fixation duration, there were no significant effects of saliency, load or presentation type on either of these measures *(*all *F*<1*).* The mean distance between each fixation and the centre of the image quantifies the extent to which participants were biased to look in the centre, with lower average distances indicating a greater central bias. If the memory task affects this bias (e.g., because participants adopt a strategy of staring at the centre), then we might expect a difference in this measure between low and high loads. We did indeed find a main effect of load on participants’ distance to the centre, *F* (1, 42) = 4.759*, p =* 0.035*, η*^*2*^* =* 0.102. There were no significant effects of saliency or presentation type (both *F*<1)*,* and no load and presentation type interaction, *F* (1, 42) =1.463*, p* = 0.233, *η*^*2*^ = 0.034, or higher order interactions. This indicates that there is an increased centre bias in the high-load condition relative to the low-load condition.

**Fig. 2:**
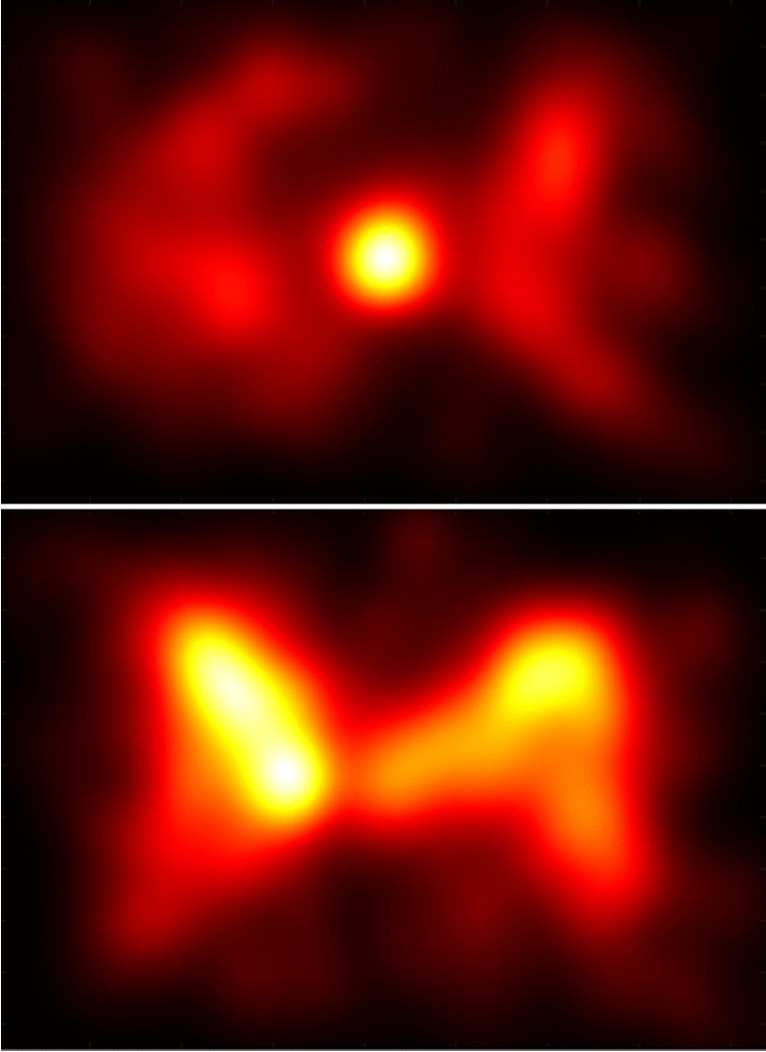
Fixation distribution across participants (pooled over sequential and simultaneous presentation) for high load (**above**) and low load (**below**). Hotter colours indicate a greater density of fixations. Note that fixations were less spread out during the high-load condition (entropy = 18.78) than during the low-load condition (entropy = 19.42)

The same difference is visible when looking at the overall fixation distribution (Fig. 2). These distributions were created by convolving the location of all fixations in each condition with a Gaussian kernel. We also generated fixation maps for each participant, and we used the heatmap entropy to quantify how spread out or exploratory the fixations were in each condition. Entropy is a measure from information theory that has often been used to quantify the dispersion of eye fixations (for a review, see Shiferaw et al., 2019). Entropy varies from a theoretical minimum of zero (if all fixations were on exactly the same pixel) to a maximum that depends on the size of the image. Distributions with higher entropy are more dispersed or less predictable. In Table 1 we report the mean (and SD) entropy in bits. There was a significant effect of load on entropy, *F* (1, 42) = 42.272*, p <* 0.001*, η*^*2*^* =* 0.502. Fixations in low-load trials were more widely dispersed across the viewing area than those in the high-load condition. There were no effects of saliency or presentation type*,* and no interactions (all *F*<1).

### Fixations to social elements

**Fig. 3:**
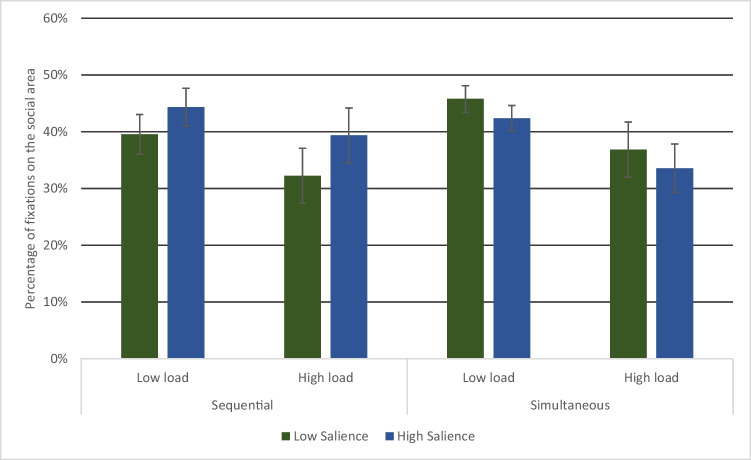
Percentage of fixations on the social area, across conditions. Bars show the mean with standard errors

Table 2 shows the proportion of fixations to the different regions of interest. It is clear from this table that, across all conditions, the social area was fixated much more often than the non-social area. We therefore examined the proportion of fixations on the social area in order to see whether memorising spatial information affects this social prioritisation effect. There was a significant effect of load, *F* (1, 42) *=* 5.156*, p =* 0.029*, η*^*2*^* =* 0.114, indicating that participants looked less often at the social object when memorising high loads of information (Fig. 3). There was no interaction between load and presentation type*, F (*1, 42*)* = 0.195*, p =* 0.661*, η*^*2*^* =* 0.005*.* There was no main effect of saliency or presentation type (both *F*<1)*,* but a marginal interaction between saliency and presentation type, *F* (1, 42*) =*3.623*, p =* 0.064*, η*^*2*^* =* 0.079*.* No other interactions were significant (all *F*<1).

**Table 2:** Percentage of fixations on each region of interest. Values show the mean across participants, with standard deviation in parentheses

	Load	High load		Low load	
	Saliency of non-social object	High	Low	High	Low
	Presentation type				
Percentage fixations on the social area	Sequential	39.37% (21.71%)	32.25% (21.47%)	44.34% (14.85%)	39.53% (15.68%)
	Simultaneous	34.51% (20.39%)	37.80% (23.37%)	43.14% (10.37%)	45.46% (11.62%)
Percentage fixations on the non-social area	Sequential	24.29% (15.01%)	18.11% (13.06%)	24.57% (18.11%)	17.24% (10.90%)
	Simultaneous	20.13% (13.96%)	18.73% (13.08%)	23.32% (12.67%)	23.96% (11.22%)

### Fixations to non-social elements

For this analysis, we considered the proportion of fixations on the non-social. In particular, we reasoned that higher loads might make people more susceptible to capture by salient objects, resulting in a larger difference between high and low salient objects in the high-load condition. There was a trend towards an effect of saliency, *F* (1, 42) = 3.695*, p =* 0.061*, η*^*2*^* =* 0*.*081*,* with high salient objects fixated more often. However, there was no effect of load *(F*<1), presentation type *(F*<1), or their interaction *(F*<1). The effect of saliency was more noticeable in the sequential presentation condition, but the interaction between saliency and presentation type fell short of significance*, F (*1, 42*) =* 2.890*, p =* 0.097*, η*^*2*^* =* 0.064.

### The first fixation

In previous studies (e.g., Anderson & Donk, 2017) it has been proposed that the first fixation on a scene might reflect “early”, more automatic attentional capture. We therefore carried out an additional analysis on the landing point of the first saccade (i.e., the first fixation after leaving the centre where viewing was constrained to begin). We examined the proportion of these fixations on the social and non-social areas in each condition. For the proportion of fixations to the social object, there was an effect of load that matched the pattern seen in the overall analysis, *F (*1, 42) = 6.60, *p* = 0.014, *η*^*2*^ = 0.136. There were fewer initial fixations to the social area in the high-load condition (*M* = 36.26%, *SEM* = 4.21) than in the low-load condition (*M* = 49.25, *SEM* = 3.56). In general, it was quite likely that the first saccade was targeted towards the social region of interest. There were no significant effects of saliency (*F*<1) or presentation type (*F*<1), and no interactions. For the proportion of first fixations to the non-social object, there was a marginal effect of saliency*, F (*1, 42*) =* 3.11*, p =* 0.085*, η*^*2*^* =* 0.069. High saliency objects (*M* = 13.11%, *SEM* = 1.81) were somewhat more likely to be fixated than low saliency objects (*M* = 8.71%, *SEM* = 1.77), but these fixations were quite rare in general. There were no other significant effects on the first fixations to the non-social object (all *F*<1).

## General discussion

We asked participants to memorise spatial information while freely viewing scenes containing social and non-social items. Our aim was to examine whether the social prioritisation effect would remain undiminished in the face of competition for perceptual processing resources, or competing demands on eye movements and attention, in the two tasks. In general, observers spent much of their time fixating on the social object in our pictures, consistent with previous reports (Birmingham et al., 2009; End & Gamer, 2019; Martinez-Cedillo et al., 2022). In all conditions, the social object was fixated much more often than the non-social object. This was also the case on the very first fixation, with up to 50% of the first saccades targeting the person in the scene. In addition, we found little effect of the salience of the non-social object, suggesting that in the face of the presence of the social item, the salience of the non-social elements no longer exerts a strong effect on performance (see Birmingham et al., 2009).

In previous research (Martinez-Cedillo et al., 2022) we showed that the social prioritisation effect was immune to competition from increasing the demand imposed by a secondary cognitive task. This invulnerability to disruption by “cognitive load” contrasts with the increased interference typically seen when cognitive load is increased in the context of physically salient distractors (Boot et al., 2005; Burnham, 2010; Lavie & De Fockert, 2005). These prior results suggested that the social prioritisation effect may be automatic, in the sense that the implicit goal to attend to social elements overrides or is distinct from the control processes used to retain information in working memory.

Critically, in the present study, fixations to the social object *were* reduced when participants retained six compared to one location in memory. This is a novel effect. Although the social region was still fixated more than the non-social region, even in high-load trials, it indicates that, at least some of the time, participants were able to avoid looking at people in conditions of high load. The same effect of load was found on the first eye movement in the scene, with this eye movement being more likely to target the social object when memory load was low. The overall number of eye movements did not differ as a function of load. Instead, the distribution of eye movements between the social element and the other areas changed. One reason for this is that participants tended to look more towards the centre of the screen, and to be less exploratory, in the high-load condition (see below for a further discussion of this point).

Fixations to the non-social object were not affected by load. This is interesting because it suggests that high-load conditions did not only cause participants to look at the centre of the screen and avoid looking at anything; instead, the load effect was selectively on the social region, which implies that orienting to people requires different resources when it comes to attentional control. The effects of model-predicted saliency, meanwhile, were limited, with only a marginal difference between high and low saliency objects, even on the first saccade. The present study was not constructed to look at this in detail, and it may be that the changes in saliency here were not large enough to have an impact, or that they were masked by other confounds in the choice of stimuli. In future research it would be possible to look at the predictiveness of saliency in different load conditions, across the whole image, and with reference to more recent modelling approaches (e.g., Kümmerer et al., 2015).

The current results place some limits on the social prioritisation effect, showing certain conditions under which it is reduced. Other researchers have shown that social prioritisation can be modified by instructions that require inspecting different parts of a scene. For example, Flechsenhar and Gamer (2017) asked participants to perform different tasks such as counting blue objects or estimating the percentage of white pixels in a scene. As in the present study, people in the scenes continued to be fixated more than non-social and salient objects, even though the people were not relevant for the task. As we would expect, the bias to look at people could be reduced by a task explicitly focusing on other parts of the image. However, the role of the difficulty of the different tasks was not clear in that study. In the present experiment, in contrast, we used a secondary memory task that did not involve the inspected images and in which we could control task difficulty through load.

In the *Introduction*, we described two ways of thinking about the potential impact of a spatial memory load on the social prioritisation effect. According to load theory, the identification of visual stimuli draws on perceptual resources that are shared between competing visual stimuli and tasks. In particular, the theory suggests overlap between the resources required to complete visual and spatial working memory and perceptual tasks. It may be that under such conditions the social elements are not fully identified and therefore not tagged as social elements of particular relevance. An alternative is that the social elements are identified as social but under conditions of competition they do not draw spatial attention to their location, either due to resource limitations or because such spatial attention is being used to rehearse locations in spatial working memory (see Awh & Jonides, 2001; Smyth & Scholey, 1994).

Related to this latter possibility it could have been that those eye movements to areas identified as socially relevant are suppressed and overridden in order to prevent interference with shifts of spatial attention that serve to rehearse locations in the spatial memory task. However, arguing against this possibility we found equivalent disruption of the social prioritisation effect by both simultaneous and sequential presentation of the spatial memory stimuli. Previous research has drawn a distinction between simultaneous and sequential presentation; for example, the model of Logie and Pearson (1997) distinguishes the “visual cache”, which is concerned with the configural properties of sets of spatial locations, with the “inner scribe”, which acts to serially rehearse sets of sequentially presented locations. Indeed, empirical data from dual-task studies (e.g., Della Salla et al., 1999) and from performance in the abducted eye paradigm (Pearson et al., 2014) supports the greater involvement of shifts of the eyes and attention in supporting sequential rather than simultaneous spatial memory. That we observe equivalence between simultaneous and sequential spatial memory argues against the idea that the reduction in the social prioritisation effect observed is linked to serial spatial rehearsal.

The most parsimonious explanation of the current data is in terms of more general competition for perceptual resources leading to reduced processing of the scene. This competition had selective effects on the social objects. Pushing this explanation to its logical conclusion, it might suggest that the identification of significant social objects in scenes is not necessarily resource-free. Earlier suggestions that processing faces and highly familiar objects might be immune to increases in task-relevant perceptual load could then be traced to the use of relatively simple displays that are not characteristic of typical human environments. The picture that then emerges of the social prioritisation effect in scene viewing suggests that the effect depends on the availability of perceptual processing resources but not more general cognitive resources related to working memory. However, exactly how to explain the social prioritisation effect remains to be determined. Given that social and non-social objects do not seem to be differentially easy to categorise in scenes (e.g., Li et al., 2021, showed that both animals and vehicles could be rapidly categorised in scenes), it may be that the representations of social objects are instead tagged in memory as having high levels of importance, such that once identified they go on to have priority in controlling shifts of attention.

## Data Availability

The experiment was preregistered. Data, experimental and analysis code are available via the Open Science Framework at https://osf.io/gk5pb/
